# The impact of telework allowance and utilization on physiological and perceived stress among Swedish white-collar workers

**DOI:** 10.5271/sjweh.4234

**Published:** 2025-09-01

**Authors:** Leticia Bergamin Januario, Marina Heiden, Svend Erik Mathiassen, Gunnar Bergström, David M Hallman

**Affiliations:** 1 Centre for Musculoskeletal Research, Department of Occupational Health, Psychology and Sports Sciences, University of Gävle, Gävle, Sweden.; 2 Unit of Intervention and Implementation Research for Worker Health, Institute of Environmental Medicine, Karolinska Institutet, Stockholm, Sweden.

**Keywords:** heart rate variability, remote work, telecommuting, working from home, office work

## Abstract

**Objectives:**

We aimed to assess the impact of telework conditions on stress levels among 294 Swedish white-collar workers.

**Methods:**

Telework during the COVID-19 pandemic was evaluated in terms of the allowance to telework (ie, the degree to which the employee could decide whether to telework), and the utilization of that allowance, using self-reported questions with answers dichotomized into ‘high’ and ‘low’. Perceived stress was measured using the Single Item Stress Question and physiological stress was measured using parameters of heart rate variability (HRV) continuously for three days [root mean square of successive differences (RMSSD) and standard deviation of the interbeat intervals of normal heart beats (SDNN)]. Multilevel linear mixed models examined the effects of telework allowance and utilization on perceived stress and HRV during work, leisure and sleep.

**Results:**

High allowance was associated with higher HRV (lower stress), while a high utilization of telework was associated with higher perceived stress and lower HRV (more stress). After adjusting for age, sex, body mass index, and objectively measured physical activity, these associations became smaller and/or non-significant, with exception of high allowance still being positively associated with higher RMSSD.

**Conclusions:**

Our findings indicate that allowing employees more autonomy in telework decisions (ie, a high allowance in this study) is associated with reduced physiological stress. These results can be used by organizations to improve telework conditions (how, where and how much), while being observant that white-collar workers do not utilize increased autonomy to work extensively and for long hours outside work. Further verification, preferably using prospective designs, is needed to confirm our results.

Telework, ie, regularly performing work-related tasks outside a designated worksite, has gained popularity in organizations employing white-collar workers due to advancements in information and communication technologies (ICT) (1). Telework has been adopted in working life, typically as a flexible arrangement enabling workers to fulfil their responsibilities from various locations, such as trains, cafés, and their homes (1).

Telework has been associated with beneficial health outcomes such as increased well-being and decreased stress, but the evidence is weak (2–4). Many individual and environmental factors, such as gender, family situation, age, personal ICT literacy and type of job, appear to play a role in determining whether telework is advantageous for health or not (2, 3). At an organizational level, giving workers the flexibility to choose *where* and *how much* they telework – a sign of trust from management – has been positively associated with job performance, productivity, and motivation (5, 6). However, unfavorable outcomes of telework have also been found, such as blurred boundaries between work and non-work, work intensification, social isolation and fatigue (7, 8).

With the COVID-19 pandemic, conditions for telework changed as white-collar workers were encouraged, or even required by governmental and company policies, to telework from home to minimize the transmission of the SARS-CoV-2 virus. The health effects of teleworking also changed during this period; several studies indicate that mental health, particularly stress, was influenced by the changed telework conditions, for example the compulsory working from home (9–11).

Throughout the COVID-19 pandemic, mobility restrictions changed depending on transmission waves, and company policies allowed workers to choose whether to telework or come to the office to different extents. However, the impacts of these varying levels of allowance on stress remains unclear, including the extent of workers’ perceived control over their telework conditions, which appear to be important to health (12, 13). According to the Job Autonomy Theory (14), control over one’s work conditions is a fundamental psychological need that influences well-being. Thus, limited autonomy in the context of telework conditions may contribute to stress, particularly if workers feel a pressure to work in arrangements that do not align with their preferences. In addition, it is necessary to consider the extent to which the allowance is utilized (ie, how much workers actually teleworked), as some may have had the option to telework but chose not to use it.

A study performed during the pandemic evaluated two aspects of telework: preference and actual frequency of telework (15). This showed that workers who preferred to telework experienced less psychological distress with increased telework frequency than workers who preferred not to telework. Similarly, Heiden et al (16) found that employees teleworking more than they preferred showed worse well-being than those who teleworked less than they would like. This suggests that a high amount of telework may result in increased stress while a high allowance is potentially beneficial.

Most of the telework studies conducted during the pandemic have used self-reports when evaluating associations with health, including work-related stress. Self-reports are likely affected by workers’ perceptions of general aspects of life while living under an ongoing pandemic (11) and should therefore be interpreted with care. An alternative way of measuring stress, which does not suffer from reporting bias to the same extent, is to monitor cardiac autonomic parameters such as heart rate variability (HRV). This gives objective data regarding the psychophysiological stress response by reflecting activity of the sympathetic and parasympathetic (vagal) components of the cardiac autonomic nervous system (17–19). Some pre-pandemic studies found a higher parasympathetic activity, ie, increased HRV and lower stress levels, when workers performed telework compared to work at office (20, 21), while a study done during the pandemic found no differences in HRV metrics when comparing telework to work at office (22). However, none of these studies, and to our knowledge no other study either, have at present addressed associations between telework allowance and utilization thereof on perceived and physiological indicators of stress during the COVID-19 pandemic.

We hypothesized that workers with limited telework allowance (i.e., those explicitly required to either telework or work on-site) would exhibit higher levels of perceived stress and lower HRV indices—indicating greater physiological stress—compared to those with greater telework allowance. Additionally, we expected that workers with lower telework utilization would report lower perceived stress and exhibit higher HRV (ie, lower physiological stress) than those with higher levels of telework utilization. Understanding the associations between telework allowance and utilization on the one hand, and perceived and physiological stress on the other, is important for promoting evidence-based telework policies even in post COVID-19 pandemic. As telework continues to be a common work arrangement, these findings can guide employers and policy-makers in designing flexible telework strategies that promote employee well-being, mitigate stress, and enhance autonomy in the workplace.

## Methods

### Study design and population

This study utilized cross-sectional data from an ongoing cohort [Flexible Work: Opportunity and Challenge (FLOC) 2020–2025] that describes flexible working conditions in different organizations in Sweden, as explained in the study protocol (23). Briefly, we recruited eight organizations with flexible work arrangements, such as flex-time and trust-based working hours, for a large fraction of employees. In total, 4366 white-collar workers [58.7% men, 44.9 (standard deviation (SD) 11.4) years old] were employed at these eight organizations, and all were invited to participate in the cohort. In the present study, we included white-collar workers (ie, workers involved in desk, managerial, or administrative work) employed for ≥50% time, who had the possibility to telework according to company policies, answered the web-survey and agreed to participate in the technical measurements. Workers who had chronic diseases or who were on long-term medication were excluded from the analysis.

Data were collected during the COVID-19 pandemic between June 2020 and March 2022. All participants signed an informed consent and data collection was performed according to the Declaration of Helsinki. The Swedish Ethical Review Authority approved the study (2019-06220).

### Data collection

All 4366 workers received an invitation by email to participate in the study, containing a link to a web-based survey asking for demographic, personal and work-related information, including education (completed elementary school, completed high school, completed higher education), smoking habits (smoker, non-smoker), and if the respondent wished to participate in technical measurements of heart rate (HR) and physical activity (yes, maybe, no). If the survey was initiated but not completed, we sent weekly reminders up to three times by e-mail. Approximately half (48%) of the white-collar workers answered the web-survey and <10% (N=307) agreed to participate in the technical measurements (table 1). The majority of the 307 workers who accepted to participate in the technical measurements were women (60.7%), and they were younger [mean 43.6 (SD 10.4) years] than the total population of white-collar workers in the companies.

**Table 1:** White-collar workers by organization (org) participating in each step of the data collection.

Org	Data collection period	Workers (total)	Survey respondants	Technical measurements participants
		N (%)	N (%)	N (%)
Total		4366 (100.0)	2107 (48.3)	307 (7.0)
1	May–Jun/2020	357 (100.0)	193 (54.1)	47 (13.2)
2	Oct–Dec/2020	2375 (100.0)	929 (39.1)	133 (5.6)
3	Nov–Dec/2020	324 (100.0)	204 (63.0)	16 (4.9)
4	May–Jun/2021	61 (100.0)	42 (68.9)	5 (8.2)
5	May–Jun/2021	163 (100.0)	112 (68.7)	11 (6.7)
6	May–Jun/2021	113 (100.0)	96 (85.0)	16 (14.2)
7	Mar/2022	234 (100.0)	157 (67.1)	33 (14.1)
8	Mar/2022	739 (100.0)	374 (50.6)	46 (6.2)

Workers accepting to participate in technical measurements underwent a procedure involving continuous assessments of cardiac autonomic parameters and physical activity measurements, and an additional survey addressing conditions of telework. During this procedure, researcher and participant met in a designated place at the organization, and the researcher explained how the technical measurements would be made during the course of a week, attached the devices monitoring HR and physical activity, and asked the participants to fill in a diary providing additional information about their daily activities during the measurement week. In some cases (N=78 out of the total N of 307), the participant had this procedure done remotely, if requested by him/herself or the organization.

### Telework conditions

All 307 participants joining the technical measurements were asked about telework conditions during the COVID-19 pandemic using two questions. One focused on the allowance provided by the organization to the workers for performing telework: *“Are you currently free to choose whether you telework or not?”* and the other focused on the utilization, ie how much the individual worker utilized the allowance he/she had at the organizational level: “*To what degree do you take advantage of your opportunity to telework during the ongoing pandemic?*”. For both allowance and utilization questions, four answer alternatives were provided, which were afterwards categorized as either high (“to a very high degree” / “quite a lot”) or low (“to some degree” / “not at all”). In a smaller dataset, we found telework utilization to be strongly correlated with the self-reported number of days teleworking per week (r=0.69, P<0.01; N=192), indicating that telework utilization can be used as a proxy for the extent of telework, while also offering a more nuanced perspective on telework than simply measuring the number of telework days.

### Assessment of stress

Participants were asked to rate their perceived stress over the last month in the web-based survey preceding the technical measurements, using the Single Item Stress Question [SISQ] which ranges from 0 (not at all) to 4 (very much) (24). We then considered stress by measuring cardiac autonomic parameters using a HR monitor (Bodyguard2, Firstbeat Technologies Ltd., Jyväskylä, Finland) continuously for three days, starting at the meeting with the researcher (regardless of whether the meeting was in person or online). The monitor was attached using two electrodes on the skin, one beneath the right clavicula and the other at the left rib cage. The electrocardiogram was sampled at 1000Hz to measure beat-to-beat RR-intervals. The device was worn around the clock during daily activities and only removed when taking a shower, bathing, or swimming. During the measurement days, the participants noted in their diary (i) when they got up in the morning, (ii) when they started and stopped working, (iii) if it was a non-working day, and (iv) when they went to bed in the evening. That information was used to separate the HR data into work, leisure and sleep periods, which allowed us to investigate both the overall stress levels and changes in stress levels between time periods (25). The cardiac autonomic nervous system response varies throughout the day: during night-time, HRV indices tend to have higher values and HR complexity decreases, indicating increased parasympathetic (vagal) activity and reduced sympathetic activity, which is consistent with the body’s relaxation and recovery processes during sleep, which is less prone to be influenced by environmental and behavioral factors (26, 27).

For the present study, non-working days were excluded from further analysis. We processed the RR-intervals from consecutive 5-minute sequences during periods of work, leisure and sleep, respectively, using the *Acti4* software (28) and calculated the averages of HR (bpm) and HRV in terms of the root mean square of successive differences between normal heartbeats (RMSSD) and standard deviation of the interbeat intervals of normal heart beats (SDNN), both in milliseconds (ms) (29). All data were assessed for artifacts using an automated algorithm and visual inspection (Firstbeat SPORTS 4.7 software, Firstbeat Technologies Ltd., Jyväskylä, Finland). Periods of RR-intervals with >50% detected artifacts were removed prior to the analysis of HRV. RMSSD is mainly used to estimate vagally mediated changes in the heart, with larger values indicating a more parasympathetic and relaxed state, while SDNN is considered a clinical “gold standard” measure for assessing cardiovascular health with smaller values predicting both higher morbidity and mortality (30). Larger values of RMSSD and SDNN, indicating larger HRV, thus reflect less stress and consequently better health (30, 31).

### Inclusion of covariates

Acknowledging that several factors can impact on ratings of perceived stress and HRV metrics, we selected a number of covariates that are known to influence both of these outcomes (30, 32, 33). Age (in years) and sex (man or woman) were obtained through a list provided by the human resources department in each of the eight included organizations. Body height (cm) and weight (kg) were measured during the technical measurements procedure to calculate the body mass index (BMI, kg/m^2^). Physical activity was also measured every day, in parallel to the HR recordings, using one triaxial accelerometer (Axivity AX3, Axivity Ltd, Newcastle, UK) fixed to the skin on the front of the right thigh. Accelerometer data were processed (34) to obtain time spent in physical activity of moderate and vigorous intensity (MVPA) and expressed relative to all other behaviors while awake in an isometric log ratio (ILR), using compositional data analysis procedures [ILR = √1/2×ln (MVPA/all other behaviors)] (35).

### Statistical analysis

We used frequencies and means with SD to describe the study sample with stratification by allowance and utilization of telework. To estimate the associations of telework allowance and utilization with stress (perceived as well as measured through cardiac autonomic parameters), we ran multilevel linear mixed models (LMM) in a full-factorial design. First, we tested the effects of allowance to telework and utilization of the allowed extent of telework on perceived stress, accounting for the organization and participant as random effects. For testing the effects on HRV, we ran three LMM, one for each of the outcomes (HR in bpm, RMSSD and SDNN in ms), accounting for the participant and organization as random effects.

Since HRV was measured during different periods of the day and smaller differences between day and night can be indicative of less parasympathetic activity and thus more stress and less recovery from work (25–27), we included period of the day in the analysis. Thus, we tested the effects of allowance to telework, utilization of telework and their potential interactions with period of the day (work, leisure, and sleep) on cardiac autonomic parameters. We only included interaction terms that were statistically significant (P<0.05) in the final models. We performed Bonferroni-adjusted pairwise comparisons to analyze potential differences between leisure and work with sleep as the reference. Models were run both unadjusted and with adjustment for age, sex, BMI, and the ILR describing the extent of MVPA. We performed all tests in SPSS version 29 (IBM Corp, Armonk, NY, USA) and considered effects to be statistically significant if P<0.05.

## Results

The main characteristics of the study population are shown in table 2. From the 307 white-collar workers who participated in technical measurements, 13 were excluded from further analysis due to missing information about the allowance and/or utilization of telework. The resulting 294 workers included in further analyses (table 2) were on average 43.7 (SD 10.5) years old, mostly women (62.2%), with completed higher educational level (88.7%) and a slight overweight (BMI 25.3 [SD 3.9] kg/m^2^). Only two participants were smokers. On average, the participants had 1.3 (SD 0.5) hours of MVPA per day. Most of the workers reported having a high telework allowance (N=176, 59.9%) and a high telework utilization (N=193, 65.6%).

**Table 2:** Characteristics of the 294 participants including perceived stress, as well as cardiac autonomic parameters as an average across the day and stratified by period of the day, considering allowance and utilization of telework, stratified into high and low. [MI=number of participants with missing information; BMI=body mass index; MVPA=moderate-to-vigorous physical activity; ILR=isometric log-ratio of MVPA relative to all other behaviors; bpm=beats per minutes; RMSSD=root mean square of successive differences between normal heartbeats; SD=standard deviation; SDNN=standard deviation of the RR intervals; ms=milliseconds]

		Total population (N=294)			Allowance to telework						Utilization of telework				
					High (N=176)			Low (N=118)			High (N=193)			Low (N=101)	
		N (%)	Mean (SD)		N (%)	Mean (SD)		N (%)	Mean (SD)		N(%)	Mean (SD)		N (%)	Mean (SD)
Sex (women)		183 (62.2)			111 (63.1)			72 (61.0)			136 (70.5)			47 (46.5)	
Smoking status (smoker) (MI:4)		2 (0.7)			1 (0.6)				1 (0.8)			1 (0.5)			1 (1.0)
Age (years)			43.7 (10.5)			44.0 (10.8)			43.2 (9.9)			45.0 (9.9)			41.0 (11.0)
BMI (kg/m^2^) (MI: 8)			25.3 (3.9)			25.0 (3.9)			25.7 (3.8)			25.3 (3.9)			25.4 (3.6)
MVPA (MI: 8)															
	Hours/day		1.3 (0.5)			1.3 (0.5)			1.2 (0.5)			1.3 (0.5)			1.3 (0.4)
	ILR (MVPA/ other)		-1.68 (0.28)			-1.66 (0.27)			-1.71 (0.28)			-1.68 (0.29)			-1.68 (0.26)
Perceived stress (0–4)			2.9 (1.1)			2.3 (0.9)			2.2 (0.9)			2.3 (1.0)			2.1 (0.8)
Heart rate (bpm)															
	Leisure		77.1 (10.0)			77.2 (10.4)			77.0 (9.6)			77.5 (10.3)			76.5 (9.5)
	Work		75.1 (10.0)			75.3 (10.6)			74.7 (9.0)			75.9 (10.2)			73.5 (9.4)
	Sleep		60.7 (8.2)			60.3 (8.1)			61.2 (8.4)			61.0 (8.5)			60.0 (7.6)
RMSSD (ms)															
	Leisure		30.5 (16.1)			31.0 (16.4)			29.8 (15.6)			29.3 (15.9)			32.9 (16.2)
	Work		31.9 (17.2)			32.7 (18.8)			30.8 (14.6)			30.0 (16.4)			35.7 (18.3)
	Sleep		45.6 (28.3)			45.6 (26.8)			43.7 (23.8)			42.9 (25.0)			48.6 (26.5)
SDNN (ms)															
	Leisure		64.0 (18.7)			64.1 (18.7)			63.9 (18.9)			61.6 (18.0)			68.7 (19.4)
	Work		66.9 (19.9)			66.6 (20.6)			67.4 (19.1)			63.5 (18.7)			73.4 (20.9)
	Sleep		71.0 (24.2)			70.6 (24.0)			71.0 (23.7)			68.5 (23.7)			75.1 (23.7)

Regarding the measurements of cardiac autonomic parameters, data were collected for, in total, 1177 days for the 294 participants. Among these days, 300 (25.5%) were non-working days, which were excluded from further analysis. Therefore, each participant had measurements of cardiac autonomic parameters for, on average, 3.0 (range 1–7) days. Descriptive data for HR, RMSSD and SDNN are shown in table 2.

Linear mixed models did not show any statistically significant interactions for the cardiac autonomic parameters between period of the day and either allowance to telework or utilization of telework (supplementary material, www.sjweh.fi/article/4234, table S1). Thus, we proceeded with models containing only the main effects of allowance to telework and utilization of telework (table 3).

**Table 3:** Estimated main effects (B), significance with 95% confidence intervals (CI) of high allowance and high utilization of telework (low used as reference) on perceived stress and cardiac autonomic parameters in linear mixed models. [bpm=beats per minutes; RMSSD=root mean square of successive differences between normal heartbeats; SDNN=standard deviation of RR intervals; ms=milliseconds]. **Bold** values denote statistical significance (P<0.05).

		High allowance to telework				High utilization of telework		
		B	95% CI	P-value		B	95% CI	P-value
Unadjusted models								
	Perceived stress	-0.18	-0.44−0.07	0.17		0.27	0.01−0.54	**0.04**
	Heart rate (bpm)	-0.84	-2.15−0.47	0.21		1.23	-0.13−2.59	0.08
	RMSSD (ms)	2.86	0.23−5.47	**0.03**		-5.07	-7.79−-2.36	**<0.01**
	SDNN (ms)	2.12	-0.76−5.00	0.15		-7.23	-10.22−-4.24	**<0.01**
Adjusted models *								
	Perceived stress	-0.17	-0.43−0.09	0.21		0.27	-0.01−0.54	0.06
	Heart rate (bpm)	-0.76	-2.04−0.52	0.24		0.42	-0.94−1.77	0.55
	RMSSD (ms)	3.17	0.75−5.59	**0.01**		-1.16	-3.75−1.42	0.37
	SDNN (ms)	1.98	-0.49−4.46	0.12		-1.98	-4.63−0.67	0.14

* Models adjusted for age, sex, body mass index, and the ILR reflecting the extent of moderate to vigorous physical activity.

Unadjusted models showed that workers reporting high allowance to telework had significantly higher RMSSD (indicating less stress), compared with workers reporting low allowance. Workers reporting high utilization of telework had significantly higher perceived stress and lower HRV (RMSSD and SDNN) than those reporting low utilization (table 3). After adjustment for age, sex, BMI, and the extent of MVPA, the effect on RMSSD of high allowance to telework remained statistically significant and of similar size as in the unadjusted model. The adjusted model for high utilization of telework resulted in smaller and non-significant effects for the HRV variables (table 3), while the estimated association and the 95% confidence intervals (CI) persisted for perceived stress, although being non-significant (P=0.06).

When comparing the different periods of the day, with sleep as the reference, HR was higher and HRV was reduced during work and leisure (supplementary table S2). Bonferroni pairwise comparisons between leisure and work showed that HR was higher during leisure compared with work in both unadjusted (mean difference 2.03 bpm; 95% CI 0.12−3.93; P=0.03) and adjusted (mean difference 2.14 bpm; 95% CI 0.28−4.01; P=0.02) models, while no differences between leisure and work were found for the HRV measures.

## Discussion

We aimed to address the potential associations of telework allowance and utilization with perceived stress and physiological indicators of stress (ie, HRV). We found contrasting results for allowance and utilization, suggesting beneficial and detrimental associations with stress, respectively. However, effect sizes were small and partly explained by individual factors.

We found a consistently positive association between high telework allowance and RMSSD. RMSSD is a reliable indicator of parasympathetic activity which has been related to stress both in experimental and occupational settings (17–19). Even though the effect size of high telework allowance on RMSSD was small, it still suggests that allowance to telework can be beneficial for reducing physiological stress levels. Based on the Job Autonomy Theory (14), we speculate that a larger telework allowance may enhance perceived control, by allowing workers more flexibility to arrange their work and leisure schedules according to preferences, thus reducing exposure to external stressors, including, eg, commuting and distractions at the office. Previous research has shown that greater job autonomy is associated with lower physiological stress responses (36, 37), which is in line with studies performed during the pandemic, like the present (12, 13, 15). Even though high autonomy is essentially a positive condition, important for skill development, job satisfaction, and psychological well-being (38), some evidence suggests that too much autonomy may not be beneficial for stress-related measures (39, 40) since it may, for example, lead to blurred lines between work and private life and longer working hours (41). However, we found no negative effect of high telework allowance on stress measures.

The results regarding the utilization of telework on stress pointed in the opposite direction: high utilization of telework was associated with higher perceived stress and lower HRV (indicating higher physiological stress levels (18)), but the effects were smaller than those for telework allowance and not statistically significant after adjusting for age, sex, BMI and the extent of moderate-to-vigorous physical activity. It is possible that in the context of the pandemic, social isolation and/or more extended working hours at home could have contributed to higher stress levels accompanying more telework utilization (11, 42). Thus, it is possible that high utilization of telework may induce stress partially due to work intensification including aspects such as long-working hours, accelerated work pace and perceived time-pressure (43), but this needs to be confirmed using prospective data in larger studies. Another explanation for having less perceived stress with lower utilization of telework could be that occasional work at the office might lead to better working conditions and thus favorable cardiac autonomic parameters, eg, due to adequate tools, a designated space specific for working, and the possibility to escape a maybe crowded living space at home. This could be an explanation in Sweden since governmental authorities did not enforce as rigorous lockdown measures during the pandemic as in other countries (44).

Our study was based on data collected during the COVID-19 pandemic, which could have increased the general stress levels for all workers. However, while Swedish governmental recommendations and company policies generally left considerable autonomy to individuals to decide their own personal mobility (44), restrictions were implemented, not only with regards to work but also life in general. Thus, responses to the questions addressing work could have been affected by aspects in personal life, which may, to some extent, influence the generalizability of our findings to post-COVID circumstances. Despite this, the results can be applicable in situations where workers for other reasons than a pandemic do not perceive that they have any influence on how remote work should be practiced. An example could be that work tasks or the work environment at the regular workplace are designed based on strict schedules on when to work remotely and when to be at the workplace.

Our descriptive results indicated that workers with low telework allowance and high telework utilization had overall higher pulse and lower HRV, particularly at night, suggesting a blunted day-night variation, which may reflect reduced parasympathetic activity and impaired recovery during sleep (27). However, we did not find any significant interaction on HRV between period of the day and telework conditions, indicating that neither allowance nor utilization influenced HRV differently throughout the day.

### Methodological considerations

The main strengths of our study include measuring cardiac autonomic parameters around the clock for several days, which allows for reliable and accurate information about the physiological state of workers at and outside work (45). Another strength is the adjustment using technical measures of physical activity, considering that the MVPA level has an influence on HRV (32) and that self-reported measures may overestimate physical activity (46).

Our study also has some limitations, which should be considered when interpreting the results. One concern is a potentially limited internal validity, referring to the extent to which associations can be established between variables without the influence of confounding factors. In our case, only 294 out of 4366 workers participated in the technical measurements, raising the possibility of selection bias. For example, if participants were generally more health-oriented or had less stress at baseline than non-participants, the observed associations might not fully reflect the true relationships in a broader population. Still, we found no difference in stress levels between the study sample and all responders to the questionnaire. Also, the mean age of the sample was similar to the target population. However, the sample contained a larger proportion of women (results not shown).

The fact that the study has a cross-sectional design should also be considered when interpreting the results; as well as the lack of validation of the questions used for addressing telework allowance and utilization. The sample size may not have been sufficient to detect statistical significance for small effect sizes. Also, we did not discriminate between days working at the office and days teleworking from home in the analyses. It is possible that the work location could influence the day-to-day stress levels of the workers, including effects of the location on opportunities for recovery (47, 48). It is also possible that the period in which the data was collected affected our results (c.f. table 1), due to the different waves in the COVID-19 pandemic, and the resulting differences in mobility restrictions. To some extent we did, however, consider this issue by adding the factor ‘organization’ as a random effect in our statistical models.

### Concluding remarks

Our findings suggest that a high telework allowance, ie, letting white-collar workers decide whether to telework or work at the office, is associated with less objectively measured stress, while high telework utilization tends to be associated with high perceived stress, in both cases after adjustment for personal factors. These contrasting findings support the idea that allowing employees greater flexibility in telework decisions may have an effect in reducing physiological stress, even if this effect is not strongly reflected in perceived stress measures. In current post-pandemic times, our findings can be used at the organizational level to show that stress levels among white-collar workers may be decreased by giving them autonomy to decide their telework conditions (how, where and how much), while at the same time being observant that they do not utilize this autonomy to work extensively and for long hours outside work. These guidelines can be of value when designing teleworking arrangements in the future but need to be further verified in prospective studies.

## Supplementary material

Supplementary material
