# Supplementary material for: The impact of telework allowance and utilization on physiological and perceived stress among Swedish white-collar workers
Source: Scand J Work Environ Health. 2025 Aug 29;51(5):404–12. doi: 10.5271/sjweh.4234 (PMC12414508; doi:10.5271/sjweh.4234)
Supplement: Supplementary material [file SJWEH-51-404-S001.pdf]

Is telework allowance and utilization associated with stress indicators among white-collar workers?[1]

by Leticia Bergamin Januario, PhD,[2] Marina Heiden, PhD, Svend Erik Mathiassen, PhD, Gunnar Bergström, PhD David M Hallman, PhD

1. Supplementary material
2. Correspondence to: Leticia B Januario, Centre for Musculoskeletal Research, Department of Occupational Health, Psychology and Sports Sciences, University of Gävle, Kungsbäcksvägen 47, 801 76 - Gävle, Sweden. [E-mail: Leticia.januario@hig.se]

Table S1. Fixed effects in the unadjusted linear mixed models testing perceived stress and cardiac autonomic parameters (heart rate, RMSSD and SDNN), including interaction terms.

	Perceived stress		Heart Rate (bpm)		RMSSD (ms)		SDNN (ms)	
	F	p	F	p	F	p	F	p
Main effects								
Allowance to telework	1.54	0.21	1.20	0.27	5.62	**0.02**	1.96	0.16
Utilization of telework	4.25	**0.04**	3.26	0.07	14.96	**<0.01**	20.96	**<0.01**
Period of the day	*na*	*na*	272.20	**<0.01**	30.89	**<0.01**	7.21	**<0.01**
Interaction terms								
Allowance * Period of the day	*na*	*na*	0.40	0.67	0.30	0.74	0.01	0.99
Utilization * Period of the day	*na*	*na*	0.27	0.76	0.78	0.46	0.37	0.69

Bpm: beats per minutes. RMSSD: root mean square of successive differences between normal heartbeats. SDNN: standard deviation of the RR intervals. Bold values mark statistical significance (p<0.05). na: non-applicable.

Table S2. Estimated main effects (B) with 95% confidence intervals and p-values for cardiac autonomic parameters (heart rate, RMSSD and SDNN), using sleep as the reference.

	Leisure			Work		
	B	95% CI	p	B	95% CI	p
Unadjusted models						
Heart rate (bpm)	16.48	15.04−17.92	**<0.01**	14.45[a]	13.00−15.90	**<0.01**
RMSSD (ms)	-15.06	-18.78−-11.34	**<0.01**	-13.64	-17.43−-9.85	**<0.01**
SDNN (ms)	-7.01	-10.46−-3.56	**<0.01**	-4.15	-7.68−-0.61	**0.02**
Adjusted models						
Heart rate (bpm)	16.40	14.98−17.81	**<0.01**	14.15[a]	12.85−15.66	**<0.01**
RMSSD (ms)	-15.24	-18.83−-11.66	**<0.01**	-13.84	-17.47−-10.21	**<0.01**
SDNN (ms)	-7.03	-10.17−-3.89	**<0.01**	-4.24	-7.42−-1.06	**0.01**

Adjusted models for age, sex, BMI, and physical activity (ILRs). Bpm− beats per minutes. RMSSD− root mean square of successive differences between normal heartbeats. SDNN− standard deviation of the RR intervals. ms− milliseconds. Bold values mark statistical significance (p<0.05).
a. statistically significant pairwise comparison between leisure and work (p<0.05).
